# Exposure to 50 Hz Extremely-Low-Frequency Magnetic Fields Induces No DNA Damage in Cells by Gamma H2AX Technology

**DOI:** 10.1155/2021/8510315

**Published:** 2021-02-15

**Authors:** Ye Lv, Shuchang Chen, Bing Zhu, Hong Xu, Shanshan Xu, Weiyan Liu, Yunyun Shen, Qunli Zeng

**Affiliations:** ^1^Hangzhou Center for Disease Control and Prevention, Mingshi Road, Hangzhou City, 310021 Zhejiang Province, China; ^2^Institute of Cognitive Neuroscience and Department of Psychology, Zhejiang Sci-Tech University, Hangzhou City, 310018 Zhejiang Province, China; ^3^Bioelectromagnetics Laboratory, Zhejiang University School of Medicine, Hangzhou City, 310058 Zhejiang Province, China

## Abstract

The current results for extremely-low-frequency magnetic fields (ELF-MF) on DNA damage are still debated. A sensitive indicator and systematic research are needed to assess the effects of ELF-MF. In this study, we used *γ*H2AX as an early and sensitive molecular marker to evaluate the DNA damage effects of ELF-MF in vitro. Human amnion epithelial cells (FLs), human skin fibroblast cells (HSFs), and human umbilical vein endothelial cells (HUVECs) were exposed to 50 Hz ELF-MF at 0.4, 1, and 2 mT for 15 min, 1 h, and 24 h, respectively. After exposure, cells were subjected to *γ*H2AX immunofluorescence and western blot. The results showed no significant difference in the average number of foci per cell, the percentage of *γ*H2AX foci-positive cells, or the expression of *γ*H2AX between the sham and 50 Hz ELF-MF exposure groups (*P* > 0.05). In conclusion, 50 Hz ELF-MF did not induce DNA damage in FLs, HSFs, or HUVECs, which was independent of the intensity or duration of the exposure.

## 1. Introduction

Extremely-low-frequency magnetic fields that are generated from power lines and various consumer devices have attracted public attention for the last few decades regarding their possible adverse effects on human health. It was first reported that an increased risk of childhood leukaemia was associated with electrical wiring configurations in 1979 [1]. Since then, various epidemiological surveys on the causal relationship between different diseases, such as neurodegenerative disease and angiocardiopathy, and ELF-MF have been conducted [2–4]. In 2002, ELF-MF was classified by the International Agency for Research on Cancer (IARC) as group 2B (possibly carcinogenic to humans) on the basis of epidemiological and experimental evidence of carcinogenicity and other relevant data [2].

It is generally known that genotoxic effects are the gold standard for carcinogenicity. In addition to epidemiological results, various laboratory investigations have indicated that ELF-MF may induce DNA strand breaks in vitro, indicating genotoxic potential [5]. Researchers found that 50/60 Hz ELF-MF at different levels could exert biological effects, such as DNA damage and apoptosis, through p38 activation and other pathways, and it might decrease cell viability and disturb the oxidative balance [6–10]. It could also affect the action of other substances, such as altering cellular responses to menadione-induced DNA damage [7] and reducing the effects of oxidative stress and DNA damage induced by cisplatin [6]. In addition to a high dose having an effect, in the present study, Zendehdel et al. [10] found that ELF-MF at levels less than the American Conference of Governmental Industrial Hygienists (ACGIH) exposure limit can produce DNA strand breaks. However, the current studies on ELF-MF remain inconclusive and controversial. Although parental occupational ELF-MF exposure is a possible carcinogenic factor, other studies have recently indicated that there is no relationship between parental occupational ELF-MF exposure and childhood leukaemia by applying a comprehensive quantitative job-exposure matrix (JEM) to a large international dataset [11]. Some similar studies showed insensitivity to ELF-MF in biological cells [12]. They found no evidence that ELF-MF could cause DNA damage in vivo or in vitro, as in human lens epithelial cells or neurogenic cells [13–16]. One hour of continuous and 75 min of intermittent (15 min power field on/15 min power field off) exposure indicated that MFs at power frequency may not cause DNA damage in cardiomyocytes [14]. Regarding the joint effects of ELF-MF, some studies also did not modify the cell survival and repair process of DNA damage induced by UV-B irradiation [17]. Cell types, exposure conditions, and parameters (e.g., intermittent exposure or continuous exposure) among investigations and experimental protocols from different researchers [18] could have contributed to the controversial observations.

The evident reason for this eventual inconsistency is the strong dependence of the EMF effects on a number of physical and biological parameters, which significantly varied between studies [19]; therefore, it is necessary to optimize the experimental conditions. To make the experiments controllable and the results comparable, in this study, we chose *γ*H2AX to evaluate the genetic effects of ELF-MF. *γ*H2AX is a phosphorylated form of histone H2AX and is one of the earliest markers of DNA double-strand breaks (DSBs) [20]. There is a close connection between *γ*H2AX foci and DSBs, and the *γ*H2AX assay is capable of detecting DNA damage at levels 100-fold below the detection limit of the alkaline comet assay [21]. Therefore, *γ*H2AX immunofluorescence is a sensitive and specific method to detect DSBs [22]. In this study, we selected three different cells (FLs, HSFs, and HUVECs) from different systems or organs, including the reproductive system, endothelial system, and skin, which is the largest human organ. After ELF-MF exposure at 0.4, 1, and 2 mT for 15 min, 1 h, and 24 h, respectively, we used *γ*H2AX immunofluorescence and western blot in these cell types to investigate the effects of ELF-MF on DNA damage.

## 2. Materials and Methods

### 2.1. Exposure System

The sXc-ELF exposure system used in this study was designed by the Foundation for Information Technologies in Society (IT'IS Foundation, Zurich, Switzerland). The apparatus has an incubator consisting of two identical chambers that contain a series of Helmholtz coils to maintain the environmental conditions (37°C, 5% CO_2_) (Heraeus, Germany). During the experiments, one chamber was used for the experimental group, and the coils in it were connected in-phase to generate enhanced ELF-MF for exposure. The other was for the sham group (without ELF-MF exposure), where there was an opposite phase connection inside to generate offset ELF-MF for the sham group [15]. The ELF-MF density between 0.04 and 3.55 mT can be continuously modulated. There was a computer-based control system outside to manipulate all the experimental parameters, including the frequency of ELF-MF, exposure intensity, and exposure time. Because the air cooling system was based on two fans per coil, the temperature differences between the two chambers could be maintained at less than 0.1°C.

The reference limit for occupational exposure set by the International Commission on Non-Ionizing Radiation Protection (ICNIRP) was 1.0 mT; in this study, we chose irradiation intensities of 0.4, 1, and 2 mT.

### 2.2. Cell Culture

The cell cultures were carried out at 37°C in a 5% CO_2_ humidified atmosphere. FLs were purchased from American Type Culture Collection (ATCC, Manassas, VA, USA) and cultured in minimum essential medium (MEM, HyClone) supplemented with 10% foetal bovine serum (FBS, HyClone). HSFs were obtained from the Lawrence Berkeley National Laboratory (Berkeley, California, USA) and were cultured in *α*-minimum essential medium (*α*-MEM, Gibco) supplemented with 10% FBS. HUVECs were obtained from the Toxicology Laboratory of Zhejiang University and cultured in Roswell Park Memorial Institute 1640 (RPMI, HyClone) supplemented with 10% FBS.

### 2.3. Cell Exposure

Before exposure, cells were plated in 35 mm diameter Petri dishes (Corning, USA) at an intensity of 1 × 10^5^ cells per dish. In our experiment, the exposure groups were placed in the chamber with 50 Hz sinusoidal electromagnetic fields at densities of 0.4, 1, and 2 mT for 15 min, 1 h, and 24 h. Meanwhile, the sham groups were placed in the sham chamber without ELF-MF exposure for the same amounts of time. The positive control groups were treated with 1 *μ*M 4-nitroquinoline 1-oxide (4NQO, Sigma), a chemical that could obviously induce DNA damage. Each experiment was repeated three times, and two dishes were included in each group.

### 2.4. *γ*H2AX Immunofluorescence

After exposure, the cell dishes were collected together, and cells plated onto glass coverslips were washed with phosphate-buffered saline (PBS) and fixed with 4% paraformaldehyde immediately for 15 min at 4°C. Then, we permeabilized the cells with 0.5% Triton X-100 for 15 min at 4°C. After blocking with goat serum (Zhongshan Goldenbridge Biotechnology), the cells were incubated with a primary mouse monoclonal anti-*γ*H2AX antibody (Millipore, USA; diluted 1 : 1000) for 2 h at room temperature and then incubated with a goat-anti-mouse secondary antibody conjugated with tetramethylrhodamine isothiocyanate (TRITC) for 1 h. Thereafter, the cells were incubated with 0.1 *μ*M 4′,6-diamidino-2-phenylindole (DAPI, Sigma) to stain the cell nuclei. Finally, the coverslips were removed from the Petri dishes and mounted on glass slides. Samples were observed with an Olympus AX70 fluorescence microscope (Olympus, Tokyo, Japan). At least 200 cells were scored manually for each coverslip from 5 to 10 randomly selected observation fields in a double-blind manner. We adopted the mean number of *γ*H2AX foci per cell and the percentage of *γ*H2AX-positive cells as indicators of DNA damage. Each experiment was repeated independently three times.

### 2.5. *γ*H2AX Western Blot Analysis

After exposure to ELF-MF, cells were resuspended in buffer (20 mM Tris-Cl (pH 8.0), 150 mM NaCl, 1 mM EDTA (pH 8.0), 0.5% NP-40, 1 mM PMSF, and 1 mM phosphatase inhibitor cocktail 2) at 4°C for 10 min. After collecting the nuclei by centrifugation at 6000 × *g* for 5 min, we resuspended the nuclei in 0.1 M HCl for 10 min. The histone extracts were obtained by centrifugation at 6000 × *g* for 5 min. The histone protein samples were subjected to SDS-PAGE (15%) and transferred electrophoretically to PVDF membranes. After blocking with 5% BSA, the membrane was immunoblotted with two specific primary antibodies (goat anti-mouse *γ*H2AX antibodies, Millipore, USA, diluted 1 : 3000; goat anti-rabbit H2AX, Bioworld, USA, diluted 1 : 1000) for 2 h. After washing with TBST, *γ*H2AX was detected with M700 and R800 peroxidase-conjugated secondary antibody (LI-COR, diluted 1 : 10000) for 1 h, and the blots were visualized and analysed using an Odyssey infrared fluorescence scanning imaging system (LI-COR, USA). The gray values of the protein bands were measured using the Quantity One software.

### 2.6. Statistical Analysis

Data are presented as the mean ± SEM of three independent experiments. All statistical analyses were performed with SPSS 16.0 by one-way ANOVA and two-tailed paired Student's *t*-test between ELF-MF and sham exposure groups. In addition, *P* < 0.05 was considered to have a statistically significant difference between two groups.

## 3. Results

### 3.1. Effects of 50 Hz ELF-MF Exposure on *γ*H2AX Foci Formation in FLs, HSFs, and HUVECs

**Figure 1:**
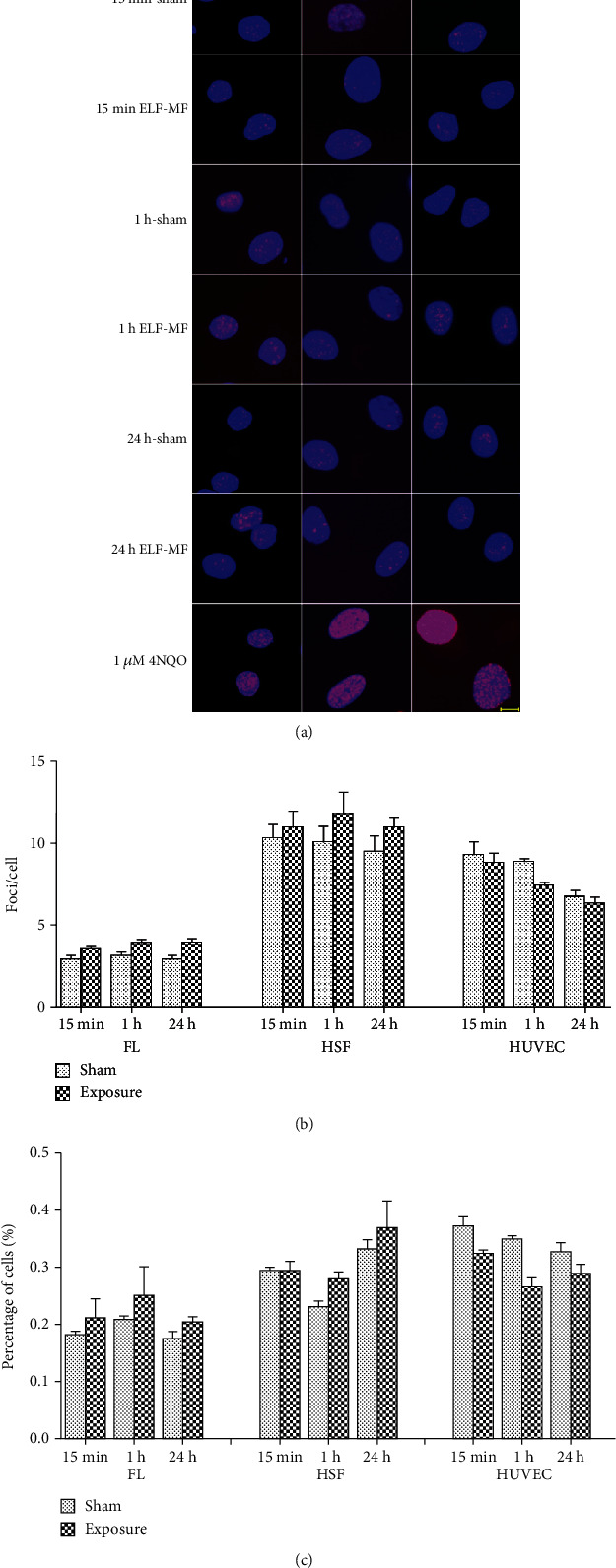
Effects of 0.4 mT ELF-MF exposure on *γ*H2AX foci formation in different cells. (a) Representative images of *γ*H2AX immunofluorescent staining after 0.4 mT ELF-MF exposure in different cells exposed for 15 min, 1 h, and 24 h; positive groups were treated with 1 *μ*M 4NQO. Nuclei were stained blue with DAPI. Scale bar, 10 *μ*m. (b) The histograms show the average numbers of *γ*H2AX foci per cell by counting over 200 cells per sample. Values are the mean ± SEM of three independent experiments. (c) The histograms show the percentage of *γ*H2AX-positive cells.

**Figure 2:**
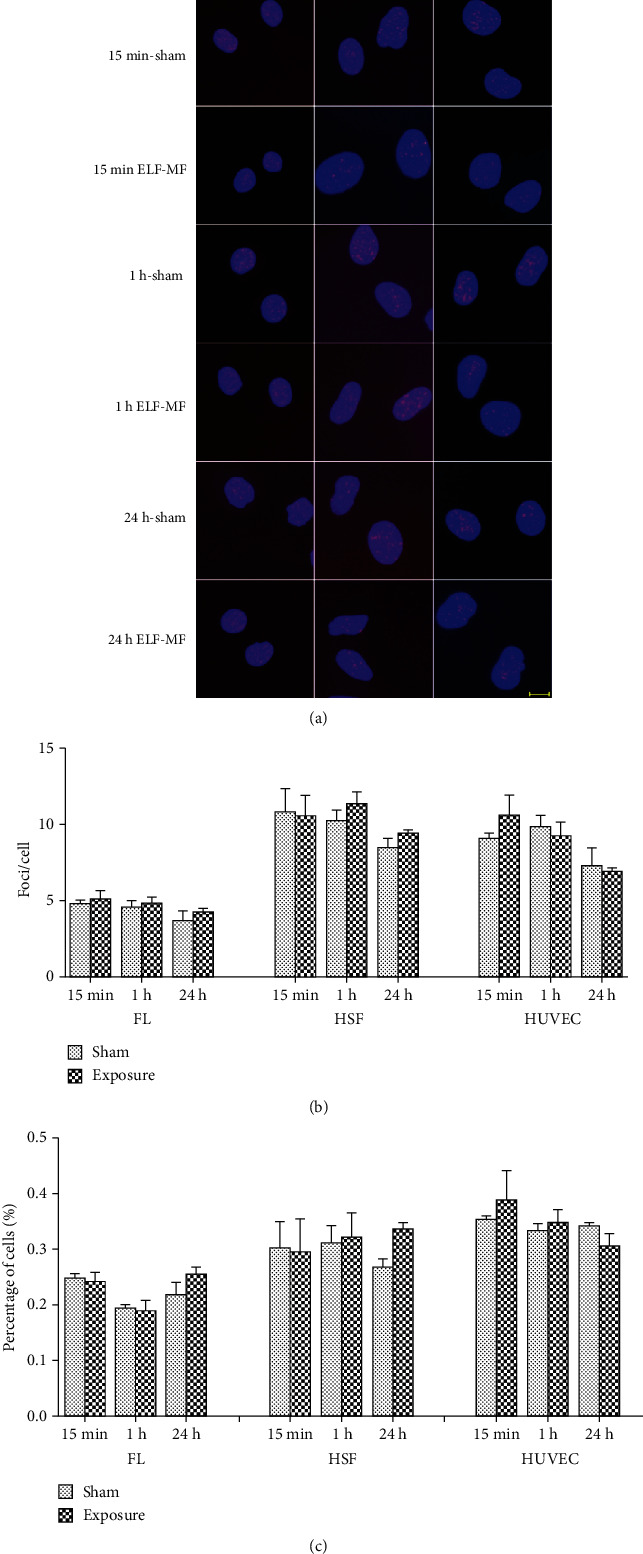
Effects of 1 mT ELF-MF exposure on *γ*H2AX foci formation in different cells. (a) Representative images of *γ*H2AX immunofluorescent staining after 1 mT ELF-MF exposure in different cells exposed for 15 min, 1 h, and 24 h. Nuclei are stained blue with DAPI. Scale bar, 10 *μ*m. (b) The histograms show the average numbers of *γ*H2AX foci per cell by counting over 200 cells per sample. Values are the mean ± SEM of three independent experiments. (c) The histograms show the percentage of *γ*H2AX-positive cells.

**Figure 3:**
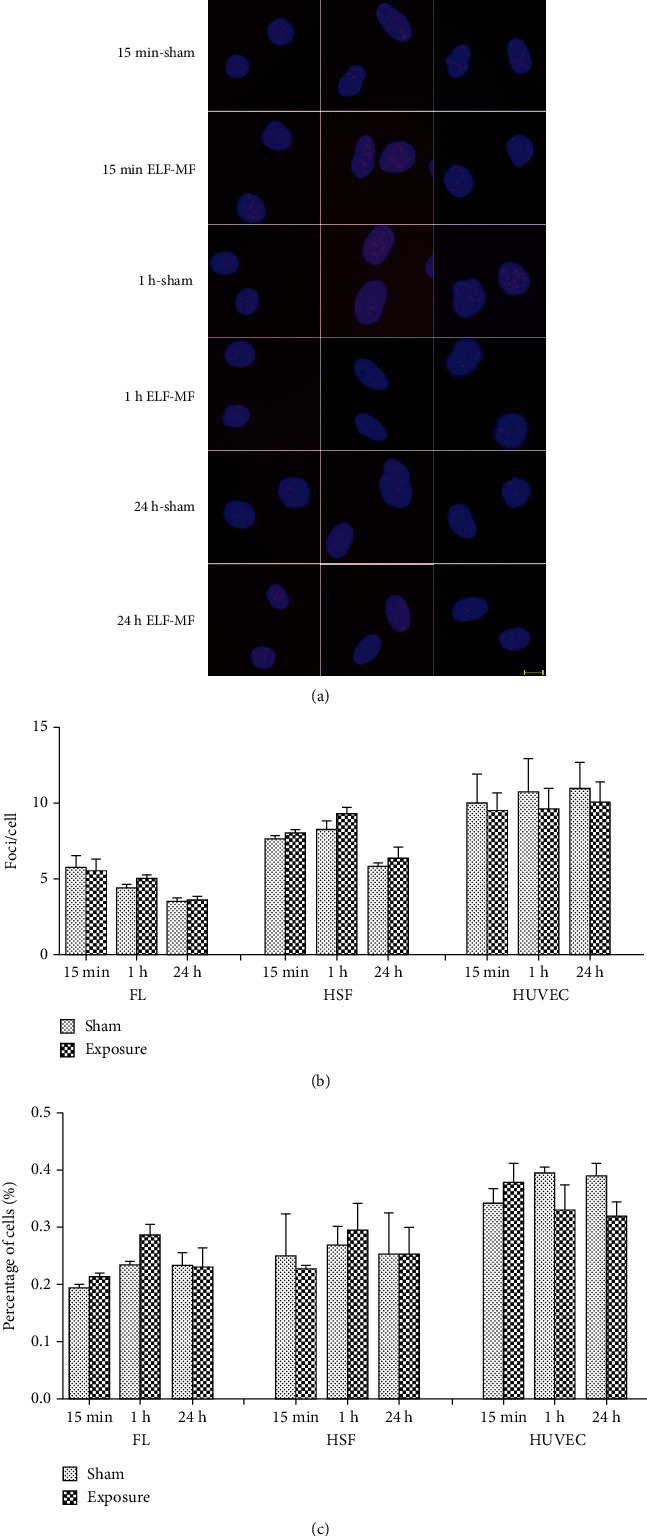
Effects of 2 mT ELF-MF exposure on *γ*H2AX foci formation in different cells. (a) Representative images of *γ*H2AX immunofluorescent staining after 2 mT ELF-MF exposure in different cells exposed for 15 min, 1 h, and 24 h. Nuclei are stained blue with DAPI. Scale bar, 10 *μ*m. (b) The histograms show the average number of *γ*H2AX foci per cell by counting over 200 cells per sample. Values are the mean ± SEM of three independent experiments. (c) The histograms show the percentage of *γ*H2AX-positive cells.

After exposure to ELF-MF for 15 min, 1 h, and 24 h at 0.4, 1, and 2 mT, respectively, the cells were subjected to immunofluorescence staining. There were no significant changes (*P* > 0.05) between the sham and ELF-MF exposure groups using the indexes of the average number of foci per cell and the percentage of *γ*H2AX foci-positive cells (Figures 1–3). However, after treatment with 1 *μ*M 4NQO for 0.5 and 1 h, there was substantial *γ*H2AX foci formation in the nuclei in all these cells. These data indicated that ELF-MF exposure did not increase FL, HSF, and HUVEC *γ*H2AX foci formation and that ELF-MF did not induce DNA damage in these three cell types.

### 3.2. Effects of 50 Hz ELF-MF Exposure on *γ*H2AX Expression in FLs, HSFs, and HUVECs

**Figure 4:**
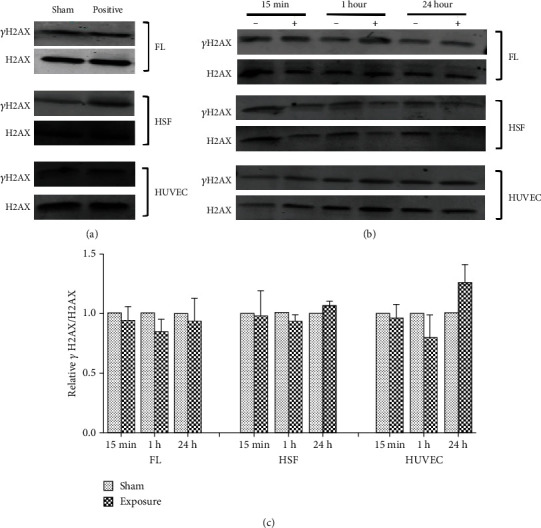
Effects of 0.4 mT ELF-MF exposure on *γ*H2AX protein expression in different cells. (a) The positive groups treated with 1 *μ*M 4NQO induced a significant increase in *γ*H2AX protein expression. (b) The cells were exposed to 0.4 mT ELF-MF for 15 min, 1 h, and 24 h. The expression of *γ*H2AX was determined by western blot. H2AX was used as a loading control. (c) Band intensities compared to the unexposed control are shown in the graph. Quantification is reflected by relative *γ*H2AX/H2AX. Values are the mean ± SEM of three independent experiments.

**Figure 5:**
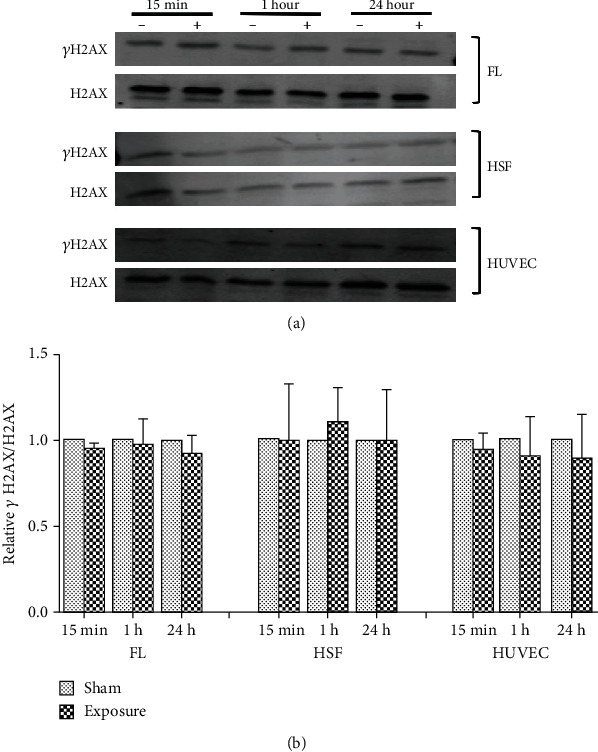
Effects of 1 mT ELF-MF exposure on *γ*H2AX protein expression in different cells. (a) The cells were exposed to 1 mT ELF-MF for 15 min, 1 h, and 24 h. The expression of *γ*H2AX was determined by western blot. H2AX was used as a loading control. (b) Band intensities compared to the unexposed control are shown in the graph. Quantification was reflected by relative *γ*H2AX/H2AX. Values are the mean ± SEM of three independent experiments.

**Figure 6:**
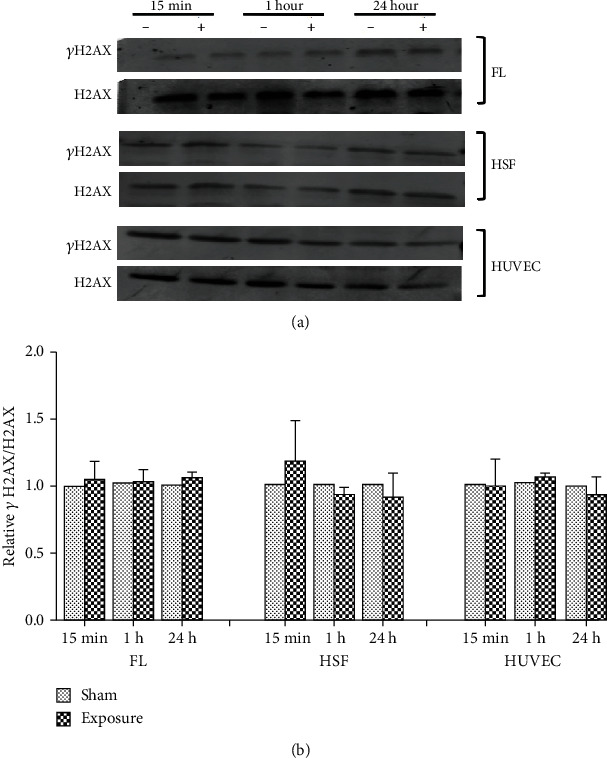
Effects of 2 mT ELF-MF exposure on *γ*H2AX protein expression in different cells. (a) The cells were exposed to 2 mT ELF-MF for 15 min, 1 h, and 24 h. The expression of *γ*H2AX was determined by western blot. H2AX was used as a loading control. (b) Band intensities compared to the unexposed control are shown in the graph. Quantification was reflected by relative *γ*H2AX/H2AX. Values are the mean ± SEM of three independent experiments.

To confirm these results, after exposure, we observed *γ*H2AX protein expression by western blot. The results showed no significant changes (*P* > 0.05) between the sham and ELF-MF exposure groups (Figures 4–6). However, there were increased expression levels of *γ*H2AX in the positive control groups that were treated with 1 *μ*M 4NQO for 0.5 and 1 h. These data indicated that ELF-MF exposure did not increase FL, HSF, and HUVEC *γ*H2AX foci expression and that ELF-MF did not induce DNA damage in these three cell types.

## 4. Discussion

In this study, we evaluated the DNA damage effects of 50 Hz ELF-MF in three different biological systems of cells by *γ*H2AX immunofluorescence and *γ*H2AX western blot. The data showed that neither *γ*H2AX foci formation nor *γ*H2AX protein expression was changed in these three cell lines. ELF-MF did not induce DNA damage in this study.

Epidemiological and experimental studies have been performed to investigate the cellular effects of ELF-MF, but the conclusions have been controversial. It is difficult to duplicate the present results due to the differences in the exposure system, field parameters, experimental design, biological systems, and related factors. Ivancsits et al. investigated the possibility of cell type-dependent genotoxicity and showed that human fibroblasts and human melanocytes are related to intermittent ELF-MF (50 Hz sinusoidal, 1 mT), but other cell types did not [23]. While the same parameters were used in this study, researchers [24] did not repeat Ivancsits et al.'s results. Moreover, Ivancsits et al. [5, 25] indicated DNA damage exposure to ELF-MF at an intensity lower than recommended by the International Commission on Non-Ionizing Radiation Protection (ICNIRP). They found that continuous or intermittent 0.02-1 mT ELF-MF (5 min on/10 min off) could increase DSBs of diploid fibroblasts, which were dependent on the magnetic flux density. In addition, Cho et al. [26] found that ELF-MF could enhance the cytotoxicity and genotoxicity of Gd in human lymphocytes.

To explore the controversial findings, we tried to investigate the induction of DNA damage by ELF-MF in different cell types at different intensities. Our previous study [15] showed that neither short-term nor long-term continuous exposure to ELF-MF could induce DNA damage in HLECs in vitro. Herein, we continued to use the same exposure scheme at intensities of 0.4, 1, and 2 mT. According to Ivancsits et al.'s study, the genetic damage of 50 Hz ELF-MF is cell-dependent [23]; therefore, we chose FLs, HSFs, and HUVECs from different systems or organs. Our present study showed that there was no significant change in *γ*H2AX by either immunofluorescence or western blot. However, we found that the baseline foci fluctuated among cell types. The average number of FLs was lower than those of HUVECs and HSFs. In addition, we found that most average numbers of FLs were near 0 (we divided the average number into four intervals: 0, 1-10, 11-20, and >20). The foci of HUVECs were generally distributed at 0 and 1-10 intervals, and the foci of HSF were distributed at 1-10 intervals. The baseline strand break was substantially higher in the HSF group than in the other two groups. This suggested that the effect of ELF-MF would be affected by the cell type, although it is not effective enough to induce the DNA damage response. The source of the cell is an important factor, and our immortalized fibroblast cell is different from Ivancsits et al.'s primary cells [23].

As an extremely weak factor, the effects of ELF-MF are subtle, and the impairment induced by ELF-MF can be repaired [4]; therefore, a sensitive method is important to detect the effects of ELF-MF. The methods of many studies were comet assays. *γ*H2AX [27] was first used for DNA damage detection induced by ionizing radiation. Because it plays a key role in assembling proteins such as Rad50, Rad51, and other repair factors to colocalize with DSBs, *γ*H2AX appears to be a good marker of DNA damage and repair [28, 29]. Defects in *γ*H2AX affect the DSB response and DNA repair [30], suggesting that *γ*H2AX is sensitive to low-intensity radiation. Moreover, *γ*H2AX foci can be observed as an effect earlier than that with the comet assay [31].

*γ*H2AX could be detected by immunofluorescence, western blot, and flow cytometry. Immunofluorescence of *γ*H2AX can directly present and be easily distinguished by nuclear staining. However, this method is also deficient because the result can be easily affected by some subjective factors, such as the visual fields of the microscope chosen in this study. In addition, the definition of positive foci can differ between technicians. To reduce the effects of subjective factors, we performed a double-blind study throughout the whole process and then verified the results by western blot.

Intermittent exposure was demonstrated to exert more severe effects on biological structures than continuous exposure. Adaptive mechanisms may be triggered when the cells are acclimatized to continuous exposure, but intermittent exposure may break the adaptation and lead to DNA damage [5, 25]. Conversely, ELF-MF could act as a coinducer of DNA damage rather than as a single genotoxic agent [3], and combined with other environmental factors, it could act in another way to investigate the biological effects of ELF-MF.

In conclusion, our results revealed that ELF-MF did not induce FL, HSF, or HUVEC DNA damage, regardless of low or high intensity or short or long exposure. More experiments with more cell types are needed to refine the exposure thresholds, frequency dependence, and dose response. The mechanism of ELF-MF in biological tissues remains a matter of debate. Future investigations on the effects of ELF-MF exposure on various animal models are needed.

## Data Availability

The data used to support the findings of this study are available from the corresponding author upon request.
